# Metformin and dietary advice to improve insulin sensitivity and promote gestational restriction of weight among pregnant women who are overweight or obese: the GRoW Randomised Trial

**DOI:** 10.1186/s12884-016-1161-z

**Published:** 2016-11-21

**Authors:** Jodie M. Dodd, Rosalie M. Grivell, Andrea R. Deussen, Gustaaf Dekker, Jennie Louise, William Hague

**Affiliations:** 1 Discipline of Obstetrics & Gynaecology, and Robinson Research Institute, The University of Adelaide, Adelaide, South Australia Australia; 2 Department of Perinatal Medicine, Women’s and Children’s Hospital, North Adelaide, South Australia Australia; 3 Department of Obstetrics & Gynaecology, Flinders Medical Centre and School of Medicine, Flinders University, Adelaide, Australia; 4 Lyell McEwin Hospital, Elizabeth Vale, South Australia Australia; 5 The University of Adelaide, School of Public Health, Adelaide, South Australia Australia; 6 Discipline of Obstetrics & Gynaecology, and Robinson Institute, Women’s & Children’s Hospital, The University of Adelaide, 72 King William Road, North Adelaide, South Australia 5006 Australia

## Abstract

**Background:**

Obesity is a significant global health problem, with approximately 50% of women entering pregnancy having a body mass index greater than or equal to 25 kg/m^2^. Obesity during pregnancy is associated with a well-recognised increased risk of adverse health outcomes both for the woman and her infant. Currently available data from large scale randomised trials and systematic reviews highlight only modest effects of antenatal dietary and lifestyle interventions in limiting gestational weight gain, with little impact on clinically relevant pregnancy outcomes. Further information evaluating alternative strategies is required.

The aims of this randomised controlled trial are to assess whether the use of metformin as an adjunct therapy to dietary and lifestyle advice for overweight and obese women during pregnancy is effective in improving maternal, fetal and infant health outcomes.

**Methods:**

Design: Multicentre randomised, controlled trial.

Inclusion Criteria: Women with a singleton, live gestation between 10^+0^-20^+0^ weeks who are obese or overweight (defined as body mass index greater than or equal to 25 kg/m^2^), at the first antenatal visit.

Trial Entry & Randomisation: Eligible, consenting women will be randomised between 10^+0^ and 20^+0^ weeks gestation using an online computer randomisation system, and randomisation schedule prepared by non-clinical research staff with balanced variable blocks. Stratification will be according to maternal BMI at trial entry, parity, and centre where planned to give birth.

Treatment Schedules: Women randomised to the *Metformin Group* will receive a supply of 500 mg oral metformin tablets. Women randomised to the *Placebo Group* will receive a supply of identical appearing and tasting placebo tablets. Women will be instructed to commence taking one tablet daily for a period of one week, increasing to a maximum of two tablets twice daily over four weeks and then continuing until birth. Women, clinicians, researchers and outcome assessors will be blinded to the allocated treatment group.

All women will receive three face-to-face sessions (two with a research dietitian and one with a trained research assistant), and three telephone calls over the course of their pregnancy, in which they will be provided with dietary and lifestyle advice, and encouraged to make change utilising a SMART goals approach.

Primary Study Outcome: infant birth weight >4000 grams.

Sample Size: 524 women to detect a difference from 15.5% to 7.35% reduction in infants with birth weight >4000 grams (*p* = 0.05, 80% power, two-tailed).

**Discussion:**

This is a protocol for a randomised trial. The findings will contribute to the development of evidence based clinical practice guidelines.

**Trial registration:**

Australian and New Zealand Clinical Trials Registry ACTRN12612001277831, prospectively registered 10^th^ of December, 2012.

## Background

Overweight and obesity represents a significant global health issue, affecting approximately 2.1 billion adults world-wide, [1] with approximately 50% of women entering pregnancy with a body mass index (BMI) in excess of 25 kg/m^2^ [2]. The risks associated with obesity during pregnancy and childbirth have been well-documented, and increase with advancing BMI [3]. Immediate pregnancy and birth complications include hypertension and pre-eclampsia, gestational diabetes, need for induction of labour, caesarean section and perinatal death, [3, 4] while infants born to women who are obese are more likely to be macrosomic, require admission to the neonatal intensive care unit, be born preterm, have a congenital anomaly, and require treatment for jaundice or hypoglycaemia [3, 4]. Increasingly, there is recognition of a longer-term health legacy, maternal obesity being associated with a greatly increased risk of both diabetes [5] and cardiovascular disease for the woman, [6] and a significant predictor of obesity in her offspring [7–9].

There is a substantial literature on gestational weight gain during pregnancy summarised by the Institute of Medicine [10]. While these recommendations advocate gestational weight gain of 7–11.5 kg for women who are overweight, and 5-9 kg for women who are obese, [10] a systematic review [11] and large-scale randomised trials [12, 13] highlight only modest effects of antenatal dietary and lifestyle interventions in limiting gestational weight gain, with little impact on clinically relevant pregnancy outcomes [11–13]. Alternative strategies during pregnancy to improve health outcomes for women who are overweight or obese, and their infants, are required.

Increasing maternal BMI is a well-recognised risk factor for the development of gestational diabetes, [3, 4] with the two conditions creating a similar metabolic environment, characterised by insulin resistance, hyperglycaemia, hyperlipidaemia, and a low-grade state of chronic inflammation, all of which influence nutrient availability for fetal growth [14]. Furthermore, women who enter pregnancy overweight or obese have an increased state of insulin resistance when compared with women of normal BMI [15, 16].

Metformin is an oral biguanide, has insulin sensitising properties, and is used increasingly in the treatment of gestational diabetes [17]. Metformin inhibits both gluconeogenesis and glycogenolysis, thereby reducing hepatic glucose production, [18] while increasing insulin mediated skeletal muscle and adipocyte glucose utilisation [19]. Metformin also promotes weight loss in non-pregnant individuals [20, 21].

Given the limited effect of antenatal dietary and lifestyle interventions during pregnancy on both gestational weight gain and clinical pregnancy and birth outcomes, [11–13] there is a need for further evaluation of the role of adjuvant therapies, including metformin, as a strategy to improve health for women who are overweight or obese. The aims of this randomised trial are to evaluate the effects of antenatal metformin and dietary advice among overweight and obese pregnant women on maternal, fetal and infant health outcomes.

The primary hypothesis is that antenatal metformin and dietary advice in overweight and obese pregnant women will reduce the risk of an infant with birth weight greater than 4000 grams.

The secondary hypotheses are that antenatal meltformin and dietary and lifestyle advice in overweight and obese pregnant women will

- Reduce the risk of morbidity from other adverse outcomes for the infant;
- Reduce the risk of maternal morbidity from adverse outcomes;
- Improve maternal quality of life and well-being; and
- Reduce the costs of health care.

## Methods

### Study design

Multicentre randomised, placebo controlled trial.

### Study setting

Public maternity hospitals in metropolitan Adelaide including the Women’s and Children’s Hospital, The Lyell McEwin Hospital and Flinders Medical Centre.

### Inclusion criteria

Women with a singleton, live gestation between 10^+0^-20^+0^ weeks who are overweight (BMI 25.0 to 29.9 kg/m^2^) or obese (BMI ≥30 kg/m^2^), at their first antenatal visit will be eligible to participate.

### Exclusion criteria

Women with a multiple pregnancy, type 1 or 2 diabetes diagnosed prior to pregnancy, or with significant renal or hepatic impairment such that metformin would be contraindicated will be excluded from participation.

### Trial entry

Eligible women will be identified in the antenatal clinic of participating centres, given the trial information sheet and counselled by a researcher, before obtaining informed written consent. Randomisation will occur using the computer based randomisation service of the Discipline of Obstetrics and Gynaecology, The University of Adelaide.

The randomisation schedule will use balanced variable blocks, and will be prepared by an investigator not involved with recruitment or clinical care. There will be stratification of women according to parity (0 versus 1 or more), BMI at booking visit (25 to 29.9 kg/m^2^ versus ≥30 kg/m^2^), and collaborating centre. Eligible women will be randomised to either the ‘Metformin Group’ or the ‘Placebo Group’.

### Supply and blinding of study medication

Metformin tablets 500 mg and placebo tablets identical in appearance will be packed by an independent pharmaceutical packaging company (Pharmaceutical Packaging Professionals, Victoria) and coded according to the randomisation schedule. The women, their caregivers and research staff will be blinded to treatment allocation. Research staff obtaining outcome data will be blinded to treatment allocation. In the unexpected situation where a clinician directly involved in the care of an individual woman considers it essential to ongoing clinical care that treatment group allocation be revealed, this will be performed by an individual not directly involved in the day-to-day management of the trial, and conveyed directly to the clinician.

### Treatment schedules

Women who are randomised to the *Metformin Group* will receive a 16 week supply of oral metformin tablets 500 mg, and a further 12 week supply at 28 weeks of their pregnancy. Women will be instructed to commence taking tablets from the time of randomisation, starting with one tablet daily for a period of one week, increasing to a maximum of two tablets twice daily (maximum dose 2000 mg daily) over four weeks as tolerated and then continuing until birth.

Women who are randomised to the *Placebo Group* will receive a 16 week supply of identical appearing placebo tablets, and a further 12 week supply at 28 weeks of their pregnancy. Women will instructed to commence taking tablets from the time of randomisation, starting with one tablet daily for a period of one week, increasing to a maximum of two tablets twice daily over four weeks as tolerated and then continuing until birth.

Treatment compliance will be assessed via completion of a short questionnaire at 36 weeks’ gestation in which women will be asked whether they have taken the medication during the previous month. Women who respond ‘no’ will be considered non-compliant with the treatment, and will be asked to indicate why they are not taking the study medication. All women will be asked if they have experienced symptoms consistent with known side effects of metformin.

### Ongoing follow-up of all women in both treatment groups

Over the course of pregnancy, each woman will receive three face-to-face sessions (two with the dietitian shortly after trial entry and again at 28 weeks gestation, and one with a research assistant at 36 weeks gestation) and three telephone calls from the research assistant at 20, 24, and 32 weeks gestation. The intervention has been designed to maximise flexibility and choice for women, while providing advice that can be incorporated into each individual’s lifestyle.

Dietary advice provided will be consistent with current Australian dietary standards, [22] and based on our experience with the LIMIT randomised trial [12, 23]. The dietary intervention will maintain a balance of carbohydrates, fat and protein, while specifically encouraging women to reduce their intake of energy dense and non-core foods high in refined carbohydrates and saturated fats, while increasing their intake of fibre, and promoting consumption of two serves of fruit, five serves of vegetables, and three serves of dairy each day [12, 22, 23].

Tailoring of the intervention will be informed by stage theories of health decision making that propose that individuals progress through a series of cognitive phases when undertaking behavioural change [24]. Initially, there will be a planning session with a research dietitian, in which women will be provided with written dietary and activity information, an individual diet and physical activity plan, recipe book and example menu plans. Women will be encouraged to set achievable goals for dietary and exercise change, supported to make these lifestyle changes and to self-monitor their progress, using a SMART goals approach. Women will be encouraged to involve their partner or significant support person in these sessions. These principles will be reinforced at subsequent face-to-face visits with the dietitian and research assistant, and during the telephone contacts [12, 23].

All women will be asked to complete a food frequency questionnaire, exercise diary, and quality of life assessments at trial entry, 28 and 36 weeks gestation, and six months postpartum. Their weight will be recorded at trial entry, at 28 weeks gestation, and at 36 weeks gestation or nearest to birth. All women will be encouraged to attend for a research ultrasound at 28 and 36 weeks gestation that does not constitute routine clinical care, to monitor fetal growth, and in particular the development of small for gestational age infants.

For women who are diagnosed with gestational diabetes, it will be assumed that they are receiving metformin, and further metformin or insulin will be added as required to maintain appropriate glycaemic control as determined by her health care provider. All other care during pregnancy and birth will be according to the practices of the hospital at which the woman is booked to give birth.

After birth, information will be obtained relating to birth and infant outcomes from the case notes by the research assistant and the delivery form completed. Similarly, the postnatal and neonatal forms will be completed for each live born infant after discharge from hospital.

See Table 1 for an outline of timing enrolment, intervention and assessments.

**Table 1:** Timeline of main activities from recruitment to the end of the clinical trial

		Eligibility screen	Informed consent	Allocation	Commence study medication	Dietary review	Weight	Questionnaires	Fetal ultrasound	Glucose tolerance test (GTT)	Baby measurements	Pregnancy and birth outcomes
	Pre-randomisation	✓	✓				✓					
	Randomisation			✓								
During Pregnancy	As soon as practicable after randomisation				✓	✓		✓				
	28 weeks					✓		✓	✓	✓		
	36 weeks					✓	✓	✓	✓			
	As soon as practicable after birth										✓	
	Six weeks after birth											✓

### Study endpoints

The primary trial outcome is the incidence of infants with birth weight >4000 grams.

The secondary study outcomes are:

1. Adverse outcomes for the infant including preterm birth (birth before 37 weeks gestation); mortality (either stillbirth (intrauterine fetal death after trial entry and prior to birth), or infant death (death of a live born infant prior to hospital discharge, and excluding lethal congenital anomalies)); infant birth weight <2500 grams; infant birth weight >4500 grams; infant birth weight >90^th^ centile for gestational age and infant sex; infant birth weight <10^th^ centile for gestational age and infant sex; hypoglycaemia requiring intravenous treatment; admission to neonatal intensive care unit, or special care baby unit; hyperbilirubinaemia requiring phototherapy; nerve palsy; fracture; birth trauma; shoulder dystocia.
2. Maternal weight gain outcomes including total gestational weight gain and gestational weight gain classified as below/within/above IoM recommendations [10].
3. Maternal changes in diet and physical activity as measured by questionnaires completed by the woman at trial entry, 28 and 36 weeks gestation, and six and 18 months after birth (Harvard Semi-quantitative Food Frequency Questionnaire, [25, 26] and the Short Questionnaire to Assess Health-enhancing physical activity (SQUASH) [27]).
4. Adverse outcomes for the woman including maternal hypertension and pre-eclampsia (in accordance with recognised Australasian Society for the Study of Hypertension in Pregnancy criteria) [28]; maternal gestational diabetes [29]; need for and length of antenatal hospital stay; antepartum haemorrhage requiring hospitalisation; preterm prelabour ruptured membranes; chorioamnionitis; need and reason for induction of labour; any antibiotic use during labour; caesarean section; postpartum haemorrhage (blood loss ≥600 mL); perineal trauma; wound infection; endometritis; length of postnatal hospital stay; thromboembolic disease; maternal death.
5. Maternal quality of life and emotional wellbeing as measured by questionnaires completed by the woman at trial entry, 28 and 36 weeks of pregnancy and six months postpartum relating to quality of life (as measured using the SF12 Health Survey Questionnaire); [30] preferences for treatment and satisfaction with care (at 6 months postpartum only); anxiety (as measured by the Short Form Spielberger State Trait Inventory [31]) and depression (as measured by the Edinburgh Postnatal Depression Scale [32]). Women will be asked a series of questions about their satisfaction with the intervention, using items modified from a previous childbirth questionnaire [33].
6. Fetal growth and wellbeing at 28 and 36 weeks’ gestation assessed by ultrasound (fetal biometry, estimated weight, liquor volume, umbilical artery Doppler waveform, and adiposity) [34].
7. Costs of health care: The primary measure of outcome for the economic analysis will be the cost per live birth. Resource use will include the provision of the dietary intervention and direct costs of health care (expected average clinic fees, the frequency and duration of GP and antenatal visits, as well as in-patient admissions), determined by hospital out-patient visits, in-patient admissions and published data sets including PBS, MBS and Australian Refined Diagnosis Related Groups (AR-DRG) cost weights. Mean costs and effectiveness between treatment groups will be compared and incremental cost effectiveness ratios (ICERs) and confidence intervals presented. For varying threshold values of cost effectiveness, acceptability curves will be presented as described previously [35]. An assessment of the sensitivity of the results to variation in measured resource use, effectiveness and/or unit costs will be undertaken using appropriate one-way and multi-way sensitivity analysis, as described previously [35].

Women will be asked to provide consent to the collection of bio-specimens (including blood/serum at trial entry, 28 weeks gestation and 36 weeks gestation, and cord blood) for potential use in future ancillary studies.

### Sample size

The primary clinical endpoint is the incidence of infants born with birth weight >4000 grams, with an estimated incidence in women eligible for this trial of 15.5% [12]. To detect a difference from 15.5% to 7.35% (alpha 0.05; power 80%), and accounting for 5% rate of attrition (based on our experience with the LIMIT Study), [12] we will recruit a total of 524 women. This sample size will be powered (80%) to detect the differences in secondary outcomes as detailed in the table below.

**Table:** 

Outcome	Difference in Incidence	Difference Detected
Caesarean Section	39.1% to 27.2%	12%
Induction of labour	36.8% to 25.1%	12%
Pre-Eclampsia	14.1% to 6%	8%
Gestational Diabetes	10% to 3.5%	6.5%

### Data management

All information obtained from this study will remain strictly confidential. While the results of the study will be published, no data will be presented to allow the identification of individual women. Data will be stored in a locked filing cabinet or on password protected computer file, and accessible only by the study team.

We have previous experience recruiting women for lifestyle intervention trials in this setting [12] and will consider adding further sites as required. Women who withdraw from ongoing participation after randomisation will be asked for consent to utilise the data already collected prior to withdrawal, including birth outcomes where possible.

### Analysis and reporting of results

The initial analysis will examine baseline characteristics of all randomised women, as an indication of comparable treatment groups, and include maternal age, parity, race, height, weight, smoking history, past obstetric history, and previous gestational diabetes. Primary and secondary outcomes will be analysed on an “intention to treat” basis, according to treatment allocation at randomisation; multiple imputation by the fully conditional specification (chained equations) method will be used to impute missing outcome data relative risks and 95% confidence intervals will be reported for the major outcomes, and the number needed to treat to benefit or harm calculated. Analyses will be adjusted for stratification variables, and for prespecified prognostic factors identified as potential confounders for particular outcomes. More specific details relating to the proposed analyses, including the handling of missing data will be outlined in the statistical analysis plan to be developed for the trial.

As the lead investigator, JMD will have access to the data, and acts as guarantor to the final data set. The trial manager (ARD) and statistician (JL) will have access to the data, and the statistician (JL) will be responsible for the development of the statistical analysis plan, prior to the conduct of any statistical analyses.

Following completion of the relevant statistical analyses, manuscripts will be prepared for publication, with subsequent presentation of results at relevant scientific and clinical meetings.

### Ethics approval

Approval to conduct this study has been obtained from the Women's and Children's Health Network (WCHN) Human Research Ethics Committee's (HREC) and local institutional approval at Women’s and Children’s Hospital, Flinders Medical Centre and the Lyell McEwin Hospital. Any amendments to the trial protocol will be notified in writing to each of the responsible HREC’s, research assistants, and clinicians involved in the study, and where relevant an amendment made to the trial registration.

A data and safety monitoring board (DSMB) will monitor efficacy (or futility) and adverse effects. Based on these considerations, the DSMB may recommend that the protocol be modified or that the GRoW trial be terminated. The DSMB will consist of experts in relevant obstetrics, neonatology and research methodology. All adverse events involving women and infants enrolled in the trial will be reviewed by a multidisciplinary committee, who will be blinded to treatment allocation, in order to clarify cause. These data will be made available for the Data Safety Monitoring Board.

## Discussion

This is a protocol for a randomised trial assessing whether the use of metformin in pregnancy, as an adjuvant therapy to dietary and lifestyle advice for women who are overweight or obese is effective in improving maternal, fetal and infant health outcomes. The findings of this trial will contribute to the currently available literature regarding the effect of metformin in pregnancy for women who are overweight and obese, [36, 37] and to the development of evidence based clinical practice guidelines.
